# PEIPNet: Parametric Efficient Image-Inpainting Network with Depthwise and Pointwise Convolution

**DOI:** 10.3390/s23198313

**Published:** 2023-10-08

**Authors:** Jaekyun Ko, Wanuk Choi, Sanghwan Lee

**Affiliations:** Department of Mechanical Convergence Engineering, Hanyang University, Seoul 04763, Republic of Korea; rhworbs1124@hanyang.ac.kr (J.K.); dhksdnr2003@hanyang.ac.kr (W.C.)

**Keywords:** image inpainting, generative adversarial networks (GANs), lightweight architecture, conditional normalization, dilated convolution, dense block, self-attention

## Abstract

Research on image-inpainting tasks has mainly focused on enhancing performance by augmenting various stages and modules. However, this trend does not consider the increase in the number of model parameters and operational memory, which increases the burden on computational resources. To solve this problem, we propose a **P**arametric **E**fficient Image **I**n**P**ainting **Net**work (PEIPNet) for efficient and effective image-inpainting. Unlike other state-of-the-art methods, the proposed model has a one-stage inpainting framework in which depthwise and pointwise convolutions are adopted to reduce the number of parameters and computational cost. To generate semantically appealing results, we selected three unique components: spatially-adaptive denormalization (SPADE), dense dilated convolution module (DDCM), and efficient self-attention (ESA). SPADE was adopted to conditionally normalize activations according to the mask in order to distinguish between damaged and undamaged regions. The DDCM was employed at every scale to overcome the gradient-vanishing obstacle and gradually fill in the pixels by capturing global information along the feature maps. The ESA was utilized to obtain clues from unmasked areas by extracting long-range information. In terms of efficiency, our model has the lowest operational memory compared with other state-of-the-art methods. Both qualitative and quantitative experiments demonstrate the generalized inpainting of our method on three public datasets: Paris StreetView, CelebA, and Places2.

## 1. Introduction

Image inpainting attempts to generate masked regions with visually satisfying image structures and regional features. This task has long been a major research area in computer vision. However, rapid changes have occurred in recent years with the emergence of deep learning. For example, traditional methods [1,2,3,4,5,6,7,8,9] only used known pixels for weighted replication or diffusion. However, deep learning-based approaches [10,11,12,13,14,15,16,17,18] continuously compress masked images into a dense latent space and then fill in the missing pixels by restoring high-level semantic information. The strength of deep learning-based methods is clearly demonstrated in large-hole inpainting tasks.

Various deep learning-based models have been introduced with the development of convolutional neural networks (CNNs) and generative adversarial networks (GANs) [19]. For instance, in accordance with the traditional concept, the methods in [16,17,20,21,22] integrate a patch-matching algorithm into the latent space to effectively generate pixels and produce results that are visually pleasing. To address the limitations of vanilla convolution, partial convolution (PConv) and gated convolution (GConv) were proposed in [13,23], in which valid pixels are conditioned by a mask that acts as a prior. The framework of inpainting models plays a key role in performance enhancement as well. Specifically, the methods in [24,25,26,27] adopt a two-stage inpainting architecture, with the overall structural features of the masked regions forecasted in the first stage and the final result predicted in the second stage by adopting the output of the previous step as a constraint. Typically, Edge Connect (EC) [24] recovers the missing edges in the first stage by piling up numerous residual blocks with dilated convolutional layers, which increases the receptive field of the model and extracts global features. In the second stage, the restored edge map is employed in texture synthesis to ensure the visual unity of the final result. However, these approaches often require separate training procedures, meaning the end-to-end training strategy cannot be applied and increasing the training complexity. Moreover, because two distinct models are employed to form a generator, the number of parameters and model complexity increase, as does the likelihood of overfitting problems. This increases the operational memory usage, which hinders the use inpainting models on platforms with restricted computational resources. For instance, models with high capacity, i.e., smartphones and embedded boards, where demand is growing rapidly, may encounter difficulties in real-world application deployment.

To solve these problems, we propose the **P**arametric **E**fficient Image **I**n**P**ainting **Net**work (PEIPNet) with a one-stage inpainting framework. First, inspired by [28], we adopt depthwise and pointwise convolutions instead of vanilla convolution to minimize the number of model parameters. Second, we employ spatially-adaptive denormalization (SPADE) [29] to conditionally normalize the feature maps according to the mask. Third, inspired by [30], we introduce a dense dilated convolution module (DDCM) to gradually fill in the missing regions at different scales and mitigate the vanishing gradient problem. Finally, efficient self-attention (ESA) [31] is utilized to capture long-range information from unmasked areas in order to enhance the inpainting accuracy.

To the best of our knowledge, the proposed PEIPNet is the first method for the image-inpainting task that has less than one million model parameters while ensuring high performance. The contributions of this study are summarized as follows:

- An efficient one-stage inpainting framework with effective modules is proposed to reduce the number of model parameters and computational costs in order to mitigate overfitting problems when trained on small datasets, with potential applications in various environments.
- Qualitative and quantitative evaluations conducted on different public datasets demonstrate the excellent performance of our method in comparison with state-of-the-art models.

## 2. Related Works

### 2.1. Image Inpainting with Patch-Based Methods

Patch-based methods were first introduced for texture synthesis [1,3], then adopted in [32] for image inpainting to fill in masked areas at the image level. These approaches normally inspect and copy comparable patches from a database or uncontaminated background into missing regions according to the distance metrics between different patches, i.e., the Euclidean distance and SIFT distance [33]. Bertalmio et al. [4] merged patch-based texture combination techniques with diffusion-based dispersion under image decomposition. PatchMatch [9] was proposed to search for similar matches between image patches. Therefore, patch-based designs for image inpainting can produce sharp outputs with similar contexts. However, generating semantically plausible images through a patch-based approach remains difficult, as a high-level understanding of the images is required.

### 2.2. Image Inpainting by Deep Generative Methods

Generative models based on neural networks for image inpainting typically encode a damaged image into a latent feature. In the latent space, the masked areas are filled in at the feature level, then the feature maps are decoded and restored into an image. In recent years, studies based on deep generative models have yielded promising results. Context Encoder (CE) [10] was one of the first neural networks to adopt deep feature learning and adversarial training [19]. CE can generate visually appealing outputs for semantic hole-filling. In terms of the loss function, the guidance loss was proposed in [16], which makes the feature maps produced in the decoder more similar to those of the ground truth images in the encoder. The methods in [11,24] employed dilated convolution to increase the receptive field of the model and capture the global features. A two-stage inpainting framework was utilized in [24,25,26,27] to fill in the pixels based on the constraints generated in the first stage. Instead of vanilla convolution, PConv [14] and GConv [23] were designed to eliminate the effects induced by placeholder values in the masked areas of the image. A contextual attention (CA) layer [18] was proposed to fill in the lost pixels with similar patches from undistorted areas in high-level feature maps. With regard to the CA layer, Sagong et al. [21] introduced a novel parallel extended decoder path with a modified CA module to reduce computational cost. However, minimizing the capacity of the model to increase its efficiency while solving the overfitting problem remains a challenge.

### 2.3. Conditional Normalization

A normalization layer for feature extraction is commonly applied in deep neural networks to aid the training process.

For instance, batch normalization (BN) [34] normalizes the activation maps across batch and spatial dimensions that affect generative networks. Instance normalization (IN) [35] differs from BN in that it normalizes the features only across spatial dimensions and enhances the outcomes of numerous generative tasks, such as style transformation. Layer normalization (LN) [36] normalizes activations across the channel and spatial dimensions, helping to train recurrent neural networks more stably. Group normalization (GN) [37] normalizes the features of the grouped channels of an instance, which boosts performance on specific vision tasks such as object detection.

Conditional normalization differs from the single set of affine parameters used in the aforementioned normalization approaches. Conditional normalization methods typically utilize external information to learn multiple sets of affine parameters. Conditional IN [38], adaptive IN [39], conditional BN [40], and SPADE [29] have been introduced for image synthesis tasks. In the image-inpainting task, region normalization (RN) [41] has been introduced for spatial region-wise normalization to overcome the limitations of typical feature normalization methods, which do not consider the impact of the corrupted areas of the input image during the normalization process.

### 2.4. Neural Networks with Lightweight Architecture

Lightweight neural networks have been introduced that allow model developers to select a small network corresponding to resource restrictions, i.e., latency and size. In particular, many studies have been conducted on image classification tasks with the aim of reducing the model capacity. Depthwise separable convolution was adopted in [42] and MobileNet in [28] to reduce the computation in the first few layers. In contrast, flattened networks [43] construct a network with fully factorized convolution, demonstrating the effectiveness of intensely factored models. Factorized convolution has been employed in factorized networks [44], similar to that in topological conjunctions. Afterwards, Xception [45] scaled up the depthwise separable convolution to exceed the performance of Inception-V3 [46]. SqueezeNet [47] is another remarkably small network that uses a bottleneck method to reduce the number of required parameters. Structured transform networks [48] and deepfried convnets [49] have been proposed to reduce computational costs. However, none of these strategies have been applied to scale down the model capacity in image-inpainting tasks.

## 3. Methodology

In this section, we first introduce the architecture of the proposed method and then explain the various loss functions used for training.

### 3.1. Generator Architecture

**Figure 1:**
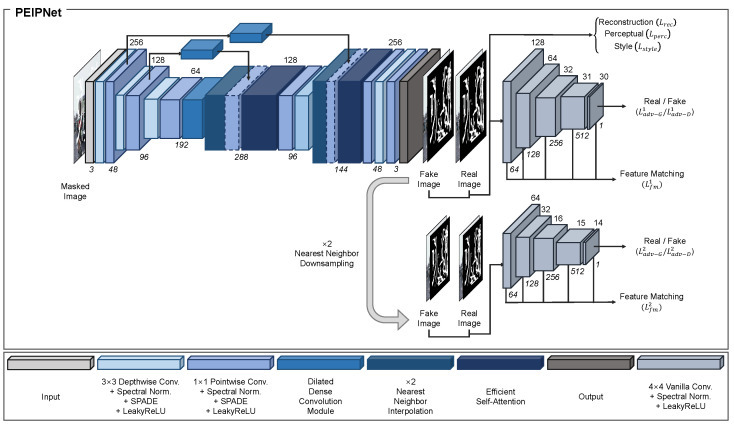
Structural overview of the PEIPNet model. PEIPNet consists of an encoder and a decoder. The encoder first compresses the damaged image into the latent space by sequentially downsampling the input by half two times. Then, the proposed DDCM fills in the masked regions using densely connected dilated convolutional layers. After the encoding process, the decoder recovers the resolution of the input image. The decoder continuously upsamples the input features two times, with the ESA generating pixels for unfilled areas by obtaining long-range information. Additionally, skip connections with DDCM are utilized to feed spatial information forward from the encoder to the decoder. At last, a multi-scale discriminator framework is used to separate fake and real images.

Figure 1 shows the architecture of the proposed method, which employs an autoencoder framework. To minimize the number of model parameters, we introduce a combination of depthwise and pointwise convolutions, as in [28]. Depthwise and pointwise convolutions are employed sequentially at the encoder and applied in the opposite order at the decoder. In both parts, depthwise convolutions with a kernel size of 3×3 are used to extract features and increase the receptive field. Pointwise convolutions with a kernel size of 1×1 are used to alter the number of channels. For all convolutional modules, spectral normalization [50] was used to stabilize the training process. SPADE [29] was chosen to conditionally normalize the masked and unmasked regions according to the semantic prior information. The SPADE framework is comprehensively analyzed in Section 3.1.1. LeakyReLU (LReLU) was chosen as the activation function, and its hyperparameter was set to 0.2.

For a given a masked input image with a size of 256×256×3, the encoder first expands the number of channels to 48. The spatial size of the feature map is then downsampled by half using a depthwise convolutional module, while the number of channels is doubled to extract a feature map of size 128×128×96. The encoder then applies the same operation again, which reduces the size of the feature map to 64×64×192. At this point, the masked regions are not yet filled with appropriate pixels. Hence, we use a DDCM to fill in the missing areas and aggregate the features with different receptive fields. The DDCM structure is thoroughly discussed in Section 3.1.2.

After passing through the encoder, the decoder recovers the resolution and generates the final output. The decoder first upsamples the input feature map using nearest-neighbor interpolation with a scaling factor of two. To obtain the spatial information, the output is concatenated channel-wise with the feature map from the encoder, which has the same spatial size. Because the masked regions in the feature map obtained by the encoder are unfilled, the DDCM is applied to the feature map immediately prior to concatenation. Moreover, we employ ESA [31] to fully extract the useful nonlocal information from the aggregated feature map. The design of ESA is described in Section 3.1.3. After ESA, the number of channels in the feature map is reduced from 288 to 96 using the pointwise and depthwise convolutional modules, yielding a feature map of size 128×128×96. The decoder uses the same procedure to extract a feature map of size 256×256×48. Finally, a pointwise convolutional module is employed to match the number of channels with the original input and produce the final result with size 256×256×3.

By adopting the aforementioned methods, the resulting PEIPNet is a lightweight architecture that can generally be used for real-world applications, i.e., embedded boards and smartphones. The model capacity and computational cost are thoroughly analyzed in Section 4.4.

#### 3.1.1. Spatially-Adaptive Denormalization

SPADE [29] was introduced as a conditional normalization method for image-to-image translation tasks. SPADE uses a semantic segmentation mask as a prior to learn the mapping function that can transform an input segmentation mask into a realistic image.

The SPADE employed in our model uses a binary mask *M* as the prior. Let $M\in L^{H\times W}$, where L is a set of integers [0,1] that denote the unmasked and masked regions and *H* and *W* are the mask height and width, respectively. Assuming the features of the *i*-th layer of a CNN with a batch of *N* samples to be $f^{i}$, the number of channels in the layer to be $C^{i}$, and the height and width of the feature map in the layer to be $H^{i}$ and $W^{i}$, respectively, the feature value at site $\left(n\in N,c\in C^{i},y\in H^{i},x\in W^{i}\right)$ is formulated as

(1) $$\gamma_{c,y,x}^{i}\left(\left[M,M^{\prime }\right]\right)⊙\frac{f_{n,c,y,x}^{i}-\mu_{c}^{i}}{\sigma_{c}^{i}}⊕\beta_{c,y,x}^{i}\left(\left[M,M^{\prime }\right]\right),$$

where $\left[M,M^{\prime }\right]$ are the set of masks, in which $M^{\prime }$ denotes the inversion of the binary mask *M*; $f_{n,c,y,x}^{i}$ is the feature at the site before normalization; $\mu_{c}^{i}$ and $\sigma_{c}^{i}$ are the mean and standard deviation of the features in channel *c*, respectively; and ⊙ and ⊕ are the element-wise multiplication and addition, respectively. Nonparametric BN [34] was employed to compute $\mu_{c}^{i}$ and $\sigma_{c}^{i}$, which are expressed as

(2) $$\mu_{c}^{i}=\frac{1}{NH^{i}W^{i}}\sum_{n,y,x}f_{n,c,y,x}^{i}$$

(3) $$\sigma_{c}^{i}=\sqrt{\frac{1}{NH^{i}W^{i}}\sum_{n,y,x}{\left(f_{n,c,y,x}^{i}-\mu_{c}^{i}\right)}^{2}}.$$

**Figure 2:**
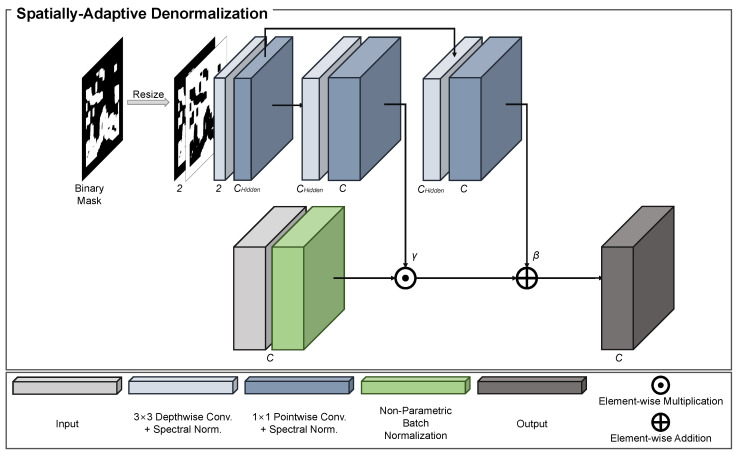
Structural overview of spatially-adaptive denormalization (SPADE). SPADE is adopted to conditionally normalize masked and unmasked regions by using the input binary mask as a prior. SPADE first normalizes the input feature using a nonparametric BN; then, the parametric variables γ and β extracted from the input binary mask are utilized to affine the normalized features.

The parametric variables $\gamma_{c,y,x}^{i}\left(\left[M,M^{\prime }\right]\right)$, and $\beta_{c,y,x}^{i}\left(\left[M,M^{\prime }\right]\right)$ in Equation (1) are the learned modulation parameters of the normalization layer. Compared with standard normalization layers, such as BN, SPADE is well suited for the image-inpainting task, as the modulation parameters adapt to the binary mask where masked and unmasked regions are given as the prior. A structural overview of the SPADE is shown in Figure 2.

Unlike SPADEs used in other previous methods, we have replaced all vanilla convolutional layers with depthwise and pointwise convolutional layers. This helps to effectively reduce the number of model parameters and computational costs while ensuring suitable performance and maintaining the overall concept of our proposal.

#### 3.1.2. Dense Dilated Convolution Module

We propose the DDCM to fill in the missing regions by combining various feature maps with different receptive fields in each stage. The main components of the DDCM are dense blocks [30] and dilated convolutional modules. We first explain the structure of the proposed DDCM, then present the reasons for its suitability in image-inpainting tasks.

A given input feature map $F_{DDCM}$ of size H×W×C passes through depthwise and pointwise convolutional modules. To reduce the number of model parameters, a bottleneck layer with a pointwise convolutional layer reduces the number of channels to $C^{\prime }$ in order to generate the feature map $F_{DDCM}^{0}$, which is expressed as

(4) $$F_{DDCM}^{0}=BL\left(PC\left(DC(F_{DDCM})\right)\right),$$

where $C^{\prime }=\frac{C}{2}$ while DC, PC, and BL denote the depthwise and pointwise convolutional module and bottleneck layer, respectively.

Then, a depthwise convolutional module with a dilation rate of two is applied along with a pointwise convolutional module to obtain the feature map $F_{DDCM}^{1}$, which is formulated as

(5) $$F_{DDCM}^{1}=PC\left(DDC_{r=2}\left(F_{DDCM}^{0}\right)\right),$$

where DDC represents the dilated depthwise convolutional module and the subscript *r* denotes the dilation rate.

The same operation with a dilation rate of four is employed to extract the feature map $F_{DDCM}^{2}$, which is defined as

(6) $$F_{DDCM}^{2}=PC\left(DDC_{r=4}\left(F_{DDCM}^{1}\right)\right).$$

Next, two feature maps $F_{DDCM}^{1}$ and $F_{DDCM}^{2}$ are concatenated channel-wise to produce $\left[F_{DDCM}^{1},F_{DDCM}^{2}\right]$ for feature aggregation. The combined feature map then passes through the bottleneck layer to decrease the number of channels from $2C^{\prime }$ to $C^{\prime }$. A depthwise convolutional module with a dilation rate of six is applied along with a pointwise convolutional module to construct the feature map $F_{DDCM}^{3}$, which is expressed as

(7) $$F_{DDCM}^{3}=PC\left(DDC_{r=6}\left(BL(\left[F_{DDCM}^{1},F_{DDCM}^{2}\right])\right)\right).$$

Using all the previous features, the same operation as in Equation (7) is then applied to yield the feature maps $F_{DDCM}^{4}$ and $F_{DDCM}^{5}$, which are formulated as

(8) $$F_{DDCM}^{4}=PC\left(DDC_{r=8}\left(BL(\left[F_{DDCM}^{1},F_{DDCM}^{2},F_{DDCM}^{3}\right])\right)\right)$$

(9) $$F_{DDCM}^{5}=PC\left(DDC_{r=10}\left(BL(\left[F_{DDCM}^{1},F_{DDCM}^{2},F_{DDCM}^{3},F_{DDCM}^{4}\right])\right)\right).$$

Finally, all the extracted feature maps are combined into one feature map to utilize the effective information. This is fed into the bottleneck layer to reduce the number of channels from $5C^{\prime }$ to *C*. Depthwise and pointwise convolutional modules are applied to produce the final output $F_{DDCM}^{{}^{\prime }}$ of size H×W×C, which is defined as

(10) $$F_{DDCM}^{{}^{\prime }}=PC\left(DC\left(BL(\left[F_{DDCM}^{1},F_{DDCM}^{2},F_{DDCM}^{3},F_{DDCM}^{4},F_{DDCM}^{5}\right])\right)\right).$$

**Figure 3:**
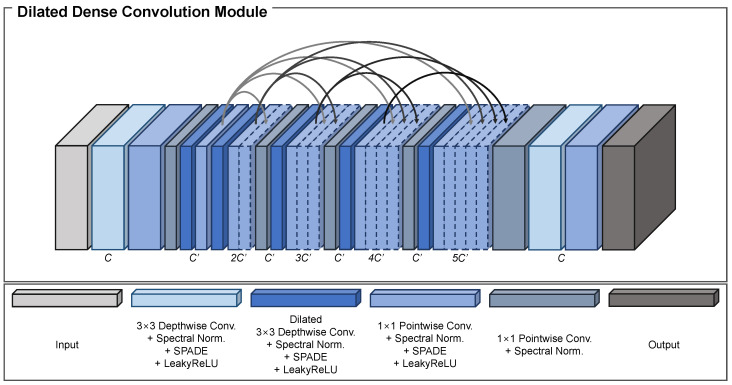
Structural overview of the dense dilated convolution module (DDCM). The DDCM consists of numerous dilated convolutional layers with different dilation rates. By adopting dilated convolution, it is possible retain a large receptive field, which is very helpful in extracting global information. The convolutional layers are connected densely in order to share features with various receptive fields. The features are aggregated with channel-wise concatenation, and pointwise convolutional layers are utilized to reduce the number of channels. Additionally, SPADE is employed at each convolutional layer for conditional normalization.

As the feature map is fed forward, the dilation rate of the depthwise convolutional module increases by two. Increasing the dilation rate expands the effective receptive field, enabling the model to look over much larger areas of the feature map. This process is applicable to image-inpainting tasks because the network can fill in the masked regions using various types of information from the entire feature map. A structural overview of the DDCM is shown in Figure 3.

The dense block was first introduced in [30] to mitigate the vanishing gradient problem, enhance feature propagation, and stimulate the reuse of the feature map. For the DDCM, we employed the dense block for two main reasons.

The first reason is to help the model converge to a better minimum point. Because the DDCMs are located in the middle of the proposed method and skip the connections between the encoder and decoder, the gradient may vanish because of the deeply stacked convolutional modules. Hence, adding a dense block provides various paths to pass the gradient at the current location along to the input, which eventually solves the vanishing gradient problem.

The second reason is to gradually fill in the missing areas in the feature map with the relevant pixels. Although the feature maps are extracted and downsampled to different scales, the missing areas are not assigned the appropriate values. Thus, we adopted a dense block to progressively generate pixels while inducing minimal artifacts. The feature maps at the front part of the DDCM (i.e., $F_{DDCM}^{1}$ and $F_{DDCM}^{2}$) are extracted using local information. Therefore, the feature maps passed along at every later stage act as the prior information, helping the model to continuously draw out features from local to global regions with an increased receptive field.

The DDCM is suitable for image-inpainting tasks for the aforementioned reasons. Moreover, as in SPADE, we have used depthwise and pointwise convolutional layers in the DDCM to minimize the model’s capacity while maintaining the concept of efficient inpainting. The effect of the DDCM is thoroughly analyzed in Section 4.7.

#### 3.1.3. Efficient Self-Attention

In our model, ESA [31] is employed to extract nonlocal information while retaining the minimum number of model parameters. In this section, we explain the details of ESA by comparing it with dot-product self-attention.

Dot-product self-attention [51] is a method for modelling long-range interactions in neural networks. Let $f^{i}\in R^{d}$ be the input features of the *i*-th layer of a CNN. Dot-product self-attention utilizes three different linear layers to transform $f^{i}$ into three feature maps: query $q^{i}\in R^{d_{k}}$, key $k^{i}\in R^{d_{k}}$, and value $v^{i}\in R^{d_{v}}$. For matrix multiplication, the queries and keys must have the same feature dimension $d_{k}$. The similarity between the *i*-th query and *j*-th key is measured by $\rho (q^{i}{k^{j}}^{T})$, where ρ denotes a normalization function. Hence, the dot-product self-attention computes the similarities between all pairs of the position in the feature maps. Utilizing the relationships as weights, the output feature is obtained using the weighted summation of the values from all positions aggregated at position *i*.

Assuming that all *n* positions’ queries, keys, and values in the matrix are formed as $Q\in R^{n\times d_{k}}$, $K\in R^{n\times d_{k}}$, and $V\in R^{n\times d_{v}}$, the output of the dot-product self-attention is defined as

(11) $$D\left(Q,K,V\right)=\rho \left(Q⊗K^{T}\right)⊗V,$$

where ⊗ represents the matrix multiplication. There are two main choices for the normalization function ρ:

(12) $$\begin{array}{c} \begin{array}{cc} Scaling:\; & \rho \left(Z\right)=\frac{Z}{n}\\ Softmax:\; & \rho \left(Z\right)=\delta_{row}\left(Z\right) \end{array} \end{array}$$

where $\delta_{row}$ is the adopted Softmax function in each row of the matrix *Z*.

The main obstacle to using this mechanism is its resource demands. This operation calculates the relationship between each pair of positions, inducing $n^{2}$ similarities. Hence, it yields a memory and computational complexity of $O(n^{2})$ and $O(d_{k}n^{2})$, respectively, where *O* denotes the large *O* notation. Owing to the resource demands, this mechanism is mainly applied to low-resolution features.

Shen et al. [31] proposed an efficient self-attention mechanism that is mathematically identical to the dot-product self-attention while being significantly faster and more memory efficient. This mechanism applies three linear layers to the input feature $f\in R^{n\times d}$ to form the queries $Q\in R^{n\times d_{k}}$, keys $K\in R^{n\times d_{k}}$, and values $V\in R^{n\times d_{v}}$. Unlike the dot-product self-attention mechanism, which solves the keys as *n* feature vectors in $R^{d_{k}}$, the efficient module considers them as $d_{k}$ single-channel feature maps. This module then generates a global context vector by utilizing each of these feature maps as weights over all areas and accumulating the value features through weighted summation.

Thus, the efficient self-attention mechanism is expressed as

(13) $$E\left(Q,K,V\right)=\rho_{q}\left(Q\right)⊗\left(\rho_{k}{\left(K\right)}^{T}⊗V\right),$$

where $\rho_{q}$ and $\rho_{k}$ denote the normalization functions for the query and key features, respectively. The two normalization approaches are formulated as

(14) $$\begin{array}{c} \begin{array}{cc} Scaling:\; & \rho_{q}\left(Z\right)=\rho_{k}\left(Z\right)=\frac{Z}{\sqrt{n}}\\ Softmax:\; & \rho_{q}=\delta_{row}\left(Z\right)\\ \; & \rho_{k}=\delta_{col}\left(Z\right) \end{array} \end{array}$$

where $\delta_{q}$ and $\delta_{k}$ are the Softmax functions for each row or column of the matrix *Z*, respectively.

The efficient self-attention is equal to the dot-product self-attention when the scaling normalization method is employed. Substituting the scaling normalization formula in Equation (12) into Equation (11) yields

(15) $$D\left(Q,K,V\right)=\frac{Q⊗K^{T}}{n}⊗V.$$

Similarly, substituting the scaling normalization formula in Equation (14) into Equation (13) produces

(16) $$E\left(Q,K,V\right)=\frac{Q}{\sqrt{n}}⊗\left(\frac{K^{T}}{\sqrt{n}}⊗V\right).$$

Because *n* is scalar and matrix multiplication is associative, Equation (16) is formulated as

(17) $$\begin{array}{c} \begin{array}{cc} E(Q,K,V) & =\frac{Q}{\sqrt{n}}⊗\left(\frac{K^{T}}{\sqrt{n}}⊗V\right)\\ \; & =\frac{1}{n}Q⊗\left(K^{T}⊗V\right)\\ \; & =\frac{1}{n}\left(Q⊗K^{T}\right)⊗V\\ \; & =\frac{Q⊗K^{T}}{n}⊗V. \end{array} \end{array}$$

**Figure 4:**
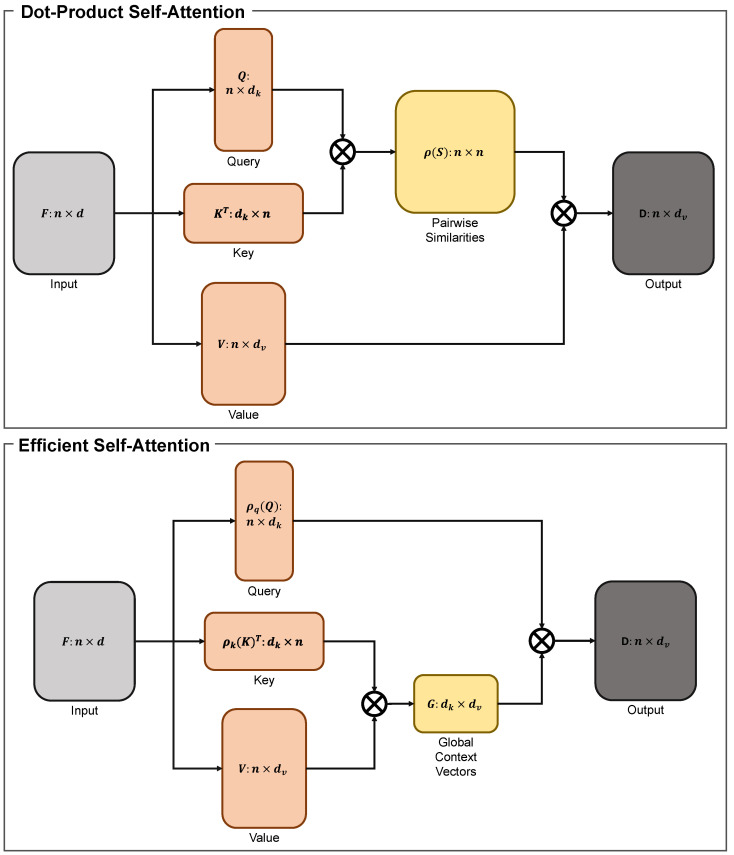
Structural overview and comparison of dot-product self-attention and efficient self-attention (ESA). Dot-product self-attention employs pairwise similarities by multiplying a query and key to extract long-range information, while ESA uses global context vectors by multiplying a key and a value. By arranging the query, key, and value in a different way, ESA permits reduced computational complexity while keeping the outputs of both methods mathematically the same.

As result, correlating Equations (15) and (17) yields E(Q,K,V)=D(Q,K,V). A structural overview of dot-product and efficient self-attention is shown in Figure 4.

Owing to the effective implementation and efficiency of the attention mechanism, we employed ESA at the decoder to extract long-range information on aggregated features and obtain clues from other areas to successfully fill in the missing areas. Furthermore, we believe that this is the first image-inpainting model that has utilized ESA. Hence, this retains the idea of a lightweight architecture, while we have modified the settings of ESA to make the model parameters even lower. The impact of ESA is examined in detail in Section 4.7.

### 3.2. Discriminator Architecture

For adversarial learning, we adopted a multiscale discriminator framework. The discriminator has a PatchGAN [52] structure with five 4×4 vanilla convolution layers and a step size of {2,2,2,1,1}. For the first three convolutional layers, the spatial size of each output feature map is halved, while the number of channels is doubled. The fourth convolutional layer doubles the number of channels, while the last convolutional layer transforms the final output feature map into a map with one channel. For all convolutional layers, spectral normalization [50] was adopted to satisfy the 1-Lipschitz constraint and ensure the training process. LReLU was used as the activation function with a hyperparameter of 0.2.

To apply a conditional setting for the learning process, the generated and target images are concatenated channel-wise with the corresponding binary mask *M* and fed into the discriminator as the input. In addition, both inputs are downsampled by half using nearest-neighbor interpolation and fed into another discriminator to satisfy the multiscale discriminator framework. The output sizes of the two discriminators are 30×30×1 and 14×14×1. A structural overview of the multiscale discriminator framework is shown in Figure 1.

### 3.3. Loss Function

To train our model, we used a combination of loss functions, namely, the adversarial loss [19], feature-matching (FM) loss [53], perceptual loss [54], style loss [55], and reconstruction loss.

First, for the adversarial loss, we employed the hinge loss [56] as the objective function for the generator, which is defined as

(18) $$\begin{array}{c} \begin{array}{cc} L_{adv-G}^{1}=-E_{\left[M,I_{out}\right]} & \left[D^{1}\left(\left[M,I_{out}\right]\right)\right]\\ L_{adv-G}^{2}=-E_{{\left[M,I_{out}\right]}_{↓}} & [D^{2}\left({\left[M,I_{out}\right]}_{↓}\right)], \end{array} \end{array}$$

where $I_{out}$ denotes the output image from the generator, ↓ is the nearest-neighbor interpolation downsampled by half, and $D^{1}$ and $D^{2}$ are discriminators with inputs of different scales. The objective functions of the two discriminators are expressed as

(19) $$\begin{array}{c} \begin{array}{c} L_{adv-D}^{1}=E_{\left[M,I_{target}\right]}\left[ReLU\left(1-D^{1}\left(\left[M,I_{target}\right]\right)\right)\right]+E_{\left[M,I_{out}\right]}[ReLU\left(1+D^{1}\left(\left[M,I_{out}\right]\right)\right)]\\ L_{adv-D}^{2}=E_{{\left[M,I_{target}\right]}_{↓}}\left[ReLU\left(1-D^{2}\left({\left[M,I_{target}\right]}_{↓}\right)\right)\right]+E_{{\left[M,I_{out}\right]}_{↓}}[ReLU\left(1+D^{2}\left({\left[M,I_{out}\right]}_{↓}\right)\right)], \end{array} \end{array}$$

where $I_{target}$ is the target image and ReLU is the rectified linear unit (ReLU) activation function.

Second, we utilized the FM loss. The FM loss causes the generator to create more reasonable and realistic outputs by measuring the features of the output image $I_{out}$ and target image $I_{target}$ in each of the discriminators. Let $S^{1}$ and $S^{2}$ be the number of convolutional layers of $D^{1}$ and $D^{2}$, respectively, let $E_{k}^{1}$ and $E_{k}^{2}$ be the number of elements in the *k*th activation layer of $D^{1}$ and $D^{2}$, respectively, and let $D_{k}^{1}$ and $D_{k}^{2}$ be the activation diagrams of layer *k* of $D^{1}$ and $D^{2}$, respectively. The FM loss is defined as

(20) $$\begin{array}{c} \begin{array}{cc} \; & L_{fm}^{1}=E\left[\sum_{k=1}^{S^{1}}\frac{1}{E_{k}^{1}}∥D_{k}^{1}\left(I_{target}\right)-D_{k}^{1}\left(I_{out}\right){∥}_{1}^{1}\right]\\ \; & L_{fm}^{2}=E\left[\sum_{k=1}^{S^{2}}\frac{1}{E_{k}^{2}}∥D_{k}^{2}\left(I_{target}\right)-D_{k}^{2}\left(I_{out}\right){∥}_{1}^{1}\right], \end{array} \end{array}$$

The mean absolute error (MAE) is adopted to compute the distance between features.

Third, we use the perceptual loss, which compares the feature maps acquired using the same convolution operation for the target and output images. This loss enables the generator to enhance the high-level semantic correlation between the two images by calculating and minimizing their differences. Specifically, we compared the distances between the activation features of the five layers (relu1-1, relu2-1, relu3-1, relu4-1, and relu5-1) of the target and generated images in the VGG-19 network [57] trained on the ImageNet dataset [58]. Thus, the perceptual loss is formulated as

(21) $$L_{perc}=E\left[\sum_{k}\frac{1}{E_{k}}∥\tau_{k}\left(I_{target}\right)-\tau_{k}\left(I_{out}\right){∥}_{1}^{1}\right],$$

where $E_{k}$ denotes the number of elements in the *k*th activation layer and $\tau_{k}$ denotes the activation diagram of the correlated layer.

Fourth, we applied the style loss, which is the correlation coefficient activation value of each activation feature channel. In our method, the VGG-19 network is used to extract feature maps, as in $L_{perc}$. Its correlation is defined by computing the eccentric covariance between the diverse activation characteristic graphs of various scales. The style loss is expressed as

(22) $$L_{style}=E_{k}\left[∥G_{k}^{\tau }\left(I_{target}\right)-G_{k}^{\tau }\left(I_{out}\right){∥}_{1}^{1}\right],$$

where *k* denotes the *k*th activation layer and $G_{k}^{\tau }$ is the Gram matrix $\tau_{k}$ with a size of $C_{k}\times C_{k}$.

Finally, we adopt the reconstruction loss to compute the distance between the corresponding pixels of the target and output images. As for the FM, perceptual, and style losses, the MAE was used to calculate the difference because it mitigates the problem of exploding gradients by maintaining a stable gradient for any input. The reconstruction loss is defined as

(23) $$L_{rec}=∥I_{target}⊙\left(1-M\right)-I_{out}⊙\left(1-M\right){∥}_{1}^{1},$$

where only the restored areas were used to compute the loss.

Using all of the abovementioned losses, the overall loss function of our model is formulated as

(24) $$\begin{array}{c} \begin{array}{cc} L_{PEIPNet}= & \lambda_{adv}\left(L_{adv-G}^{1}+L_{adv-G}^{2}\right)+\lambda_{fm}\left(L_{fm}^{1}+L_{fm}^{2}\right)+\\ \; & \lambda_{perc}L_{perc}+\lambda_{style}L_{style}+\lambda_{rec}L_{rec}, \end{array} \end{array}$$

where the loss weights were set as follows: $\lambda_{adv}=1$, $\lambda_{fm}=1\times 10^{2}$, $\lambda_{perc}=1\times 10^{2}$, $\lambda_{style}=1\times 10^{2}$, and $\lambda_{rec}=1\times 10^{3}$.

## 4. Experiments

### 4.1. Datasets

PEIPNet was trained on three public datasets: Paris StreetView [59], CelebA [60], and Places2 [61]. The Paris StreetView dataset contains 15,900 training samples and 100 testing images. The CelebA human face dataset contains approximately 160,000 training images and 19,900 testing images. The standard training dataset Places2 contains over four million images. Our model was evaluated on this validation dataset with 36,500 images. For training and testing, we followed the same procedure as in [14], in which random augmentation methods such as random translation, rotation, dilation, and cropping were used to augment the training masks. A mask set with 12,000 irregular masks that were pre-organized into six intervals according to the mask area (1∼10%, 10∼20%, ⋯, 50∼60%) was employed for testing. All the images in the Paris StreetView, CelebA, and Place2 datasets were resized to 256×256 using bicubic interpolation.

### 4.2. Compared Methods

We compared our method with state-of-the-art models such as EC [24], RFR [62], CR-Fill [63], CTSDG [64], and SPL [65]. All these models were pretrained; hence, their performance was directly evaluated in our settings.

### 4.3. Implementation Details

In the experiments, we used the Adam optimizer [66] ($\beta_{1}=0.9$, $\beta_{2}=0.999$). The learning rates were initialized to $1\times 10^{-3}$ and $4\times 10^{-3}$ for the generator and discriminators, respectively. The batch size was set to 32. Our model was trained for $1\times 10^{5}$ iterations, with the model evaluated at each $1\times 10^{3}$ iteration. The cosine annealing algorithm [67] was selected as the learning rate scheduler to slowly decay the learning rates of the generator and discriminators to $1\times 10^{-5}$ and $4\times 10^{-5}$, respectively. The PyTorch framework [68] and an NVIDIA A100 GPU with 80 GB of RAM were utilized to implement the proposed method. The official code can be found in the following link: https://github.com/JK-the-Ko/PEIPNet, accessed on 7 September 2023.

### 4.4. Analysis of Model Complexity

Because we introduced a lightweight architecture for the image-inpainting task, the number of parameters and operational memory of each model were computed and compared, as listed in Table 1. Models with a batch size of one were used to measure the memory usage. For the model capacity, our method had less than one million parameters, the smallest among the tested models. The parameter difference ratio compared with other approaches ranged from 4.5 to 57.9. For the operational memory, PEPSI [21] would be an exceptional comparison, but was excluded because the official code was not provided by its authors. Our model consumed the least amount of computational resources, i.e., approximately seven times lower compared to CR-Fill, the model with the second-lowest consumption. Having the most efficient structure in terms of both the number of parameters and operational memory provides a significant advantage in solving the overfitting problem, which commonly occurs when using a dataset with a limited number of images.

### 4.5. Qualitative Evaluations

**Figure 5:**
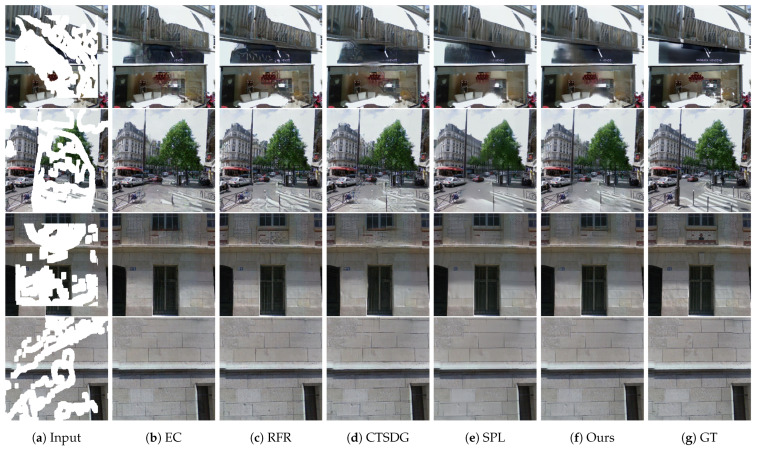
Qualitative results of our method and other models on the Paris StreetView dataset. From left to right: (**a**) input masked images, (**b**) EC [24], (**c**) RFR [62], (**d**) CTSDG [64], (**e**) SPL [65], (**f**) ours, and (**g**) ground truth images.

Figure 5 shows the qualitative comparison results on the Paris StreetView dataset. The EC model generates an edge map corresponding to the final output and identifies the global arrangement and long-range features by employing dilated convolutional layers. However, minute textural details were not extracted, resulting in an offset in the local target. For example, in the first row, the straight line above the front sign was not recovered, which corrupted the distinct boundary. In the second to fourth rows, the texture of the tree, the structure of the bricks between the middle highest window, and the pattern of the building surface were distorted. RFR sequentially fills in the missing pixels by circular feature inference, generating high-fidelity visual effects; however, serious artifacts developed as well. For example, in the first to third rows, wrinkled patterns are produced in the large masked regions. In the fourth row, checkerboard artifacts are found on the bricks, which is a common problem of transposed convolutional layers [69]. CTSDG binds the texture and structure to each other; however, the boundaries were blurred owing to the implicit usage of the structure. For instance, in the first to third rows, the straight lines are obscured, distorting the distinct boundaries of semantically different regions. In the fourth row, cross-patterned deformities developed throughout the region. SPL conducts knowledge distillation on the pretext models and modifies the features for image-inpainting. This helps in understanding the global context while providing structural supervision for the restoration of local texture. Nonetheless, the local texture was smoothed out, resulting in blurring effects. For example, although solid lines are retained in all the rows, the texture of the leaves and brick patterns are not retained in the second to fourth rows. In contrast, our proposed method restores images with a suitable balance of low- and high-level features. For all rows, the pixels were filled with clear boundaries and a semantically plausible texture, as seen in the second row. This result is attributed to the use of ESA, where the model is able to obtain hints of the texture from all areas of the corresponding feature maps.

**Figure 6:**
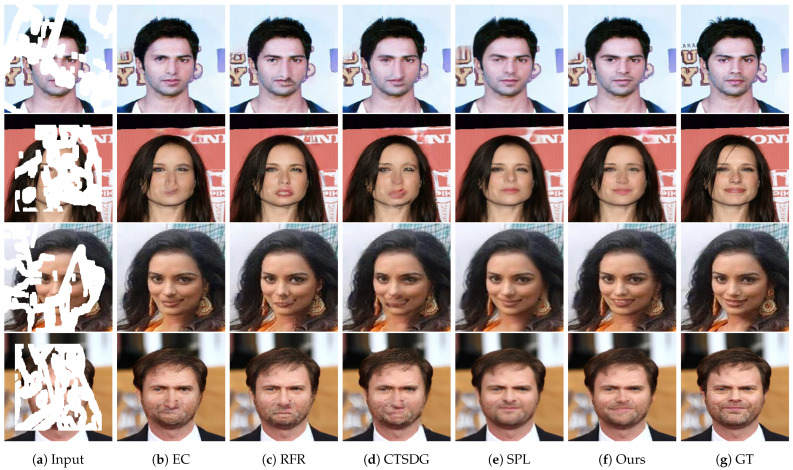
Qualitative results of our method and other models on the CelebA dataset. From left to right: (**a**) input masked images, (**b**) EC [24], (**c**) RFR [62], (**d**) CTSDG [64], (**e**) SPL [65], (**f**) ours, and (**g**) ground truth images.

Figure 6 shows the qualitative comparison results on the CelebA dataset. EC obtained unsatisfactory results, i.e., the facial structures were extremely distorted. For instance, in the first row, the position of the left eye is not symmetrical to the right eye. In the second row, the nose does not have the appropriate shape, while the mouth is barely visible. In the fourth row, although the eyes and nose have the proper silhouettes, the mouth can hardly be seen. RFR provides better results than EC, though the final outputs are not improved. Although the model generates eyes with a normal shape, the mouths in all the rows are distorted, which ruins the degree of image restoration. CTSDG had the least favorable results of all. For all rows, the facial structures are not retained due to blurring effects, and checkerboard artifacts are found in all inpainted regions. While SPL sufficiently recovered the images, there were a few implausible regions remaining. For instance, in the first row, the size of the left eye is different and relatively smaller than the right eye. In the fourth row, the wrinkles and beard on the face disappear owing to excessive smoothing. In contrast, our model generated images with the best quality. For example, in the first row, the size of the left eye is similar to that of the right eye, and is at a suitable location. In the third row, unlike the other models, our method generates a mouth with teeth, very close to the ground-truth image. In the fourth row, the wrinkles and beard with a proper mouth are retained, and there is less perceived difference compared to the other methods.

**Figure 7:**
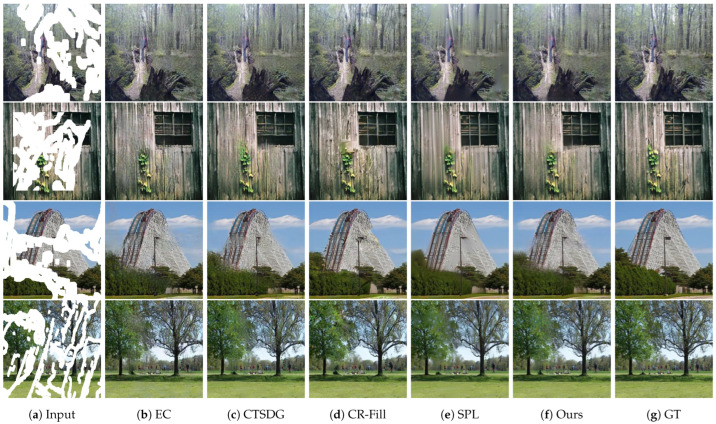
Qualitative results of our method and other models on the Places2 dataset. From left to right: (**a**) input masked images, (**b**) EC [24], (**c**) CTSDG [64], (**d**) CR-Fill [63], (**e**) SPL [65], (**f**) ours, and (**g**) ground truth images.

Figure 7 shows the qualitative comparison results on the Places2 dataset. EC restored images with an acceptable quality using an edge map; however, certain areas are not appealing. For example, in the third row, the rails of the roller coaster are connected by curved lines, which is unrealistic. In the fourth row, the leaves filled with generated pixels do not have a consistent color compared with the other regions. CTSDG produced images with indistinct boundaries, i.e., unrealistic results. For instance, in the second row, the structure of the window is not fully retained owing to the blurriness of the bottom-left region. In the third row, the ride paths appear disconnected, which is unrealistic. In the fourth row, the texture of the leaves contrasts with the other regions and is not harmonized with different areas. CR-Fill trains the generator by adopting an auxiliary contextual reconstruction task that makes the generated output more plausible, even when restored by the surrounding regions. Hence, CR-Fill reconstructed images with an acceptable quality; however, a few regions can be perceived as different. For instance, in the first and third rows, the boundaries of the trees are not obvious, and the color of the middle–right part of the ride is inconsistent. SPL produced outputs with distinct lines connecting the masked regions; however, key textures and patterns were lost owing to excessive smoothing. For example, in the first, third, and fourth rows, the textures of the objects are blurred. The generated image in the second row contains checkerboard artifacts that distort the texture and quality of the image. In contrast to other methods, our proposed model achieved a balance between the apparent boundaries and textures of various objects. For instance, all the rows have straight lines separating semantically different areas. Furthermore, the textures of the objects were effectively restored, leading to plausible results.

In summary, our proposed method effectively balances low- and high-level feature restoration. This proves the generalizability of the proposed method based on qualitative evaluations.

### 4.6. Quantitative Evaluations

To analyze the inpainting results of our proposed method and those of other models, we applied four different metrics: the Fréchet inception distance (FID) [70], learned perceptual image patch similarity (LPIPS) [71], structural similarity (SSIM), and peak signal-to-noise ratio (PSNR). The FID is a widely used quantitative metric in the field of image generation; it measures the Wasserstein-2 distance between the generated and target images utilizing a pretrained Inception-V3 model [46]. Except for the FID, the other metrics are full-reference image quality assessments, in which restored images are compared with their corresponding ground truth images. The LPIPS evaluates the restoration effect by computing the similarity between the deep features of two images using AlexNet [72]. The SSIM calculates the difference between two images in terms of their brightness, contrast, and structure. Finally, the PSNR analyzes the restoration performance by measuring the distances between the pixels of two images. The quantitative comparison results on the Paris StreetView, CelebA, and Places2 datasets are listed in Table 2, Table 3 and Table 4, respectively. For all the results, the first and second highest values are labeled in bold and underlined (↓ indicates that lower is better; ↑ indicates that higher is better).

On the Paris StreetView dataset, our PEIPNet method ranked first or second for all metrics. For mask rates of (0.1,0.2] and (0.2,0.3], PEIPNet achieved the best results, similar to the FID and LPIPS. However, for mask rates of (0.3,0.4] and (0.4,0.5], RFR had the best results, similar to the FID and LPIPS, while PEIPNet had the second-best results. For SSIM and PSNR, SPL and PEIPNet had the best and second-best results, respectively, for all mask rates. The excellent performance of PEIPNet is attributed to the low number of artifacts in the generated images. The textures of different objects were retained as well, which FID and LPIPS are highly sensitive to. Hence, PEIPNet can fill in small masked regions; however, its strength decreased on the large-hole inpainting task. This is because the DDCM and ESA encourage PEIPNet to obtain various meaningful hints from different regions of the feature maps with small masked areas by identifying global long-range and local features through dilated convolution and nonlocal attention. If there are insufficient regions from which to obtain information, the aforementioned mechanism results in reduced performance of PEIPNet.

On the CelebA dataset, PEIPNet ranked first or second for the LPIPS, SSIM, and PSNR. EC had the best outcomes for the FID with all mask rates, followed by SPL. However, the FID difference between SPL and PEIPNet was very small, except with the mask rate of (0.4,0.5]. For the LPIPS, PEIPNet had the best results with the first three mask rates and the second-best with the highest mask rate. The opposite was true for the best and second-best results of the RFR. For the SSIM and PSNR, SPL had the best values, followed by PEIPNet. As on the Paris StreetView dataset, the disparity in inpainting performance compared with the best method continued to increase as the mask rate increased, for the same reason mentioned previously.

On the Places2 dataset, PEIPNet again had either the best or second-best LPIPS, SSIM, and PSNR. Unlike on the CelebA dataset, PEIPNet had the second-best outcome for the FID with mask rates of (0.1,0.2] and (0.2,0.3]. For the LPIPS, PEIPNet had the lowest values with the first three mask rates and the second lowest with the highest mask rate; the opposite was true for SPL. For the SSIM and PSNR, PEIPNet had the second highest values for all mask rates, while SPL had the best outcomes. The same phenomenon of increased inpainting accuracy difference compared with the best result was observed on the Places2 dataset.

The proposed PEIPNet method showed exceptional performance for all metrics: FID, LPIPS, SSIM, and PSNR. In most cases, PEIPNet had the best or second-best outcome; this tendency was not observed for the other methods. Specifically, PEIPNet achieved at least the second-best results on the Paris StreetView dataset, indicating the advantage of having a small number of model parameters when training with a limited number of samples. Thus, our quantitative evaluations confirmed the generalizability of the proposed method.

### 4.7. Ablation Studies

To verify the effects of the introduced the DDCM and ESA, ablation studies using our method were conducted on the Paris StreetView dataset. Specifically, we divided the DDCM into two parts for analysis, namely, the dilated convolution and the dense block. To reduce the training time, we altered the batch size to eight for all combinations.

The quantitative results with different combinations of DDCM and ESA on the Paris StreetView dataset are listed in Table 5. For the DDCM, eliminating the entire module affected model performance; the average FID and LPIPS increased by 5.3607 and 0.0102, while the SSIM and PSNR decreased by 0.0083 and 0.4658, respectively, compared with the original model. Comparison of the two parts of the DDCM showed that applying dilated convolutional layers yielded better results for all metrics, which indicates the importance of long-range feature extraction in the image-inpainting task. ESA plays a crucial role, as the average FID and LPIPS increased by 2.32 and 0.0034, while the SSIM and PSNR decreased by 0.0025 and 0.0676, respectively. However, the decline in performance without ESA was lower than that without the DDCM, indicating its dominance in the proposed method.

**Figure 8:**
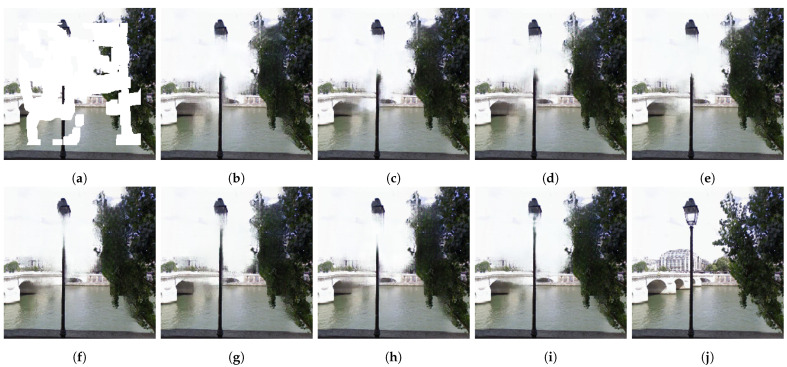
Qualitative comparison ablation experiment on the Paris StreetView dataset with different combinations of proposed DDCM and ESA. DC, DB, and ESA denote dilated convolution, dense block, and efficient self-attention, respectively. **✓** and **✗** indicate the presence of the corresponding module. (**a**) Input; (**b**) DC(**✗**)-DB(**✗**)-ESA(**✗**); (**c**) DC(**✗**)-DB(**✗**)-ESA(**✓**); (**d**) DC(**✗**)-DB(**✓**)-ESA(**✗**); (**e**) DC(**✗**)-DB(**✓**)-ESA(**✓**); (**f**) DC(**✓**)-DB(**✗**)-ESA(**✗**); (**g**) DC(**✓**)-DB(**✗**)-ESA(**✓**); (**h**) DC(**✓**)-DB(**✓**)-ESA(**✗**); (**i**) DC(**✓**)-DB(**✓**)-ESA(**✓**); (**j**) GT.

The qualitative results with different combinations of the DDCM and ESA on the Paris StreetView dataset are shown in Figure 8. Unlike the original model, the remaining combinations did not retain the streetlight structure. Specifically, the pillar was disconnected from the head of the lamp, which is unrealistic. The authentic model provided the best restoration of the texture of the leaves, demonstrating the strength of the proposed modules.

Finally, we calculated the complexities of different combinations of models, as described in Section 4.4 and summarized in Table 6. The contribution of dilated convolution was minor, with almost no change in the memory when this process was eliminated. Removing the dense block had a greater impact on the memory compared with dilated convolution, though the change remained insignificant. On the other hand, eliminating ESA had a significant impact on memory through a 4.51% reduction in the computational cost. Thus, despite its structural efficiency, adopting self-attention remains costly.

## 5. Conclusions

In this study, we have introduced the PEIPNet model for filling in missing pixels in damaged images. The proposed method consists of a one-stage inpainting framework in which depthwise and pointwise convolutions are utilized to minimize the number of parameters and computational costs. We introduce three distinct modules into the proposed model to produce semantically plausible outputs. First, SPADE was adopted to normalize the feature maps according to the input mask. Second, a DDCM was used at each scale to generate pixels and capture global information along the activations. Finally, we employed ESA to extract long-range information and obtain references from undamaged areas. As a result, PEIPNet is the first image inpainting method with less than one million model parameters, and consumes the lowest amount of operational memory among the compared state-of-the-art models. This shows the strength of our method, as it can be generally used in real-world applications which require low computational costs, i.e., smartphones and embedded boards. In addition, our experiments showed that the proposed method can be generalized in terms of both qualitative and quantitative perspectives. In future studies, we plan to reduce the model parameters and memory consumption to strengthen the main concept of lightweight architecture and real-world application. Moreover, we intend to obtain higher-resolution images for inpainting generality.

## Figures and Tables

**Table 1:** Number of parameters and complexities of compared methods. The lower the number of parameters and complexity, the more efficient the method. Our result is highlighted in **bold**.

Method	Number of Model Parameters	Operational Memory (MiB)	Parameter Difference Ratio
EC [24]	21,535,684	2291.2	23.91
RFR [62]	31,224,064	2119.5	34.66
CR-Fill [63]	4,052,478	2075.5	4.50
CTSDG [64]	52,147,787	2118.2	57.89
SPL [65]	45,595,431	2107.5	50.61
Ours	**900,875**	**301.4**	**1**

**Table 2:** Quantitative comparisons between EC, RFR, CTSDG, SPL, and our method on the Paris StreetView dataset with different mask rates. ↓ indicates that lower is better and ↑ indicates that higher is better. The best result is highlighted as **bold** and the second-best result is marked with underline.

Dataset		Paris Streetview			
**Mask Ratio**		**(0.1, 0.2]**	**(0.2, 0.3]**	**(0.3, 0.4]**	**(0.4, 0.5]**
FID (↓)	EC	18.5194	29.1657	41.9956	57.1491
	RFR	16.6533	24.3895	**34.3220**	**47.7101**
	CTSDG	18.8494	30.4578	46.1283	63.7335
	SPL	17.7120	28.6431	44.2332	58.4954
	Ours	**14.8024**	**23.5567**	38.1458	54.1898
LPIPS (↓)	EC	0.0375	0.0663	0.102	0.1479
	RFR	0.0337	0.0598	**0.0896**	**0.1302**
	CTSDG	0.0373	0.0693	0.11	0.1606
	SPL	0.0367	0.068	0.1084	0.1571
	Ours	**0.0301**	**0.0559**	0.0943	0.1428
SSIM (↑)	EC	0.9397	0.8946	0.8405	0.7764
	RFR	0.9468	0.9052	0.8533	0.7911
	CTSDG	0.9471	0.9035	0.8469	0.7826
	SPL	**0.9578**	**0.923**	**0.877**	**0.8209**
	Ours	0.9522	0.9123	0.8605	0.8012
PSNR (↑)	EC	30.9867	28.1855	25.749	23.8551
	RFR	31.76	28.866	26.2591	24.4081
	CTSDG	31.8254	28.8162	26.0823	24.2014
	SPL	**33.3091**	**30.226**	**27.3961**	**25.5072**
	Ours	32.4553	29.4006	26.6137	24.7842

**Table 3:** Quantitative comparison between EC, RFR, CTSDG, SPL, and our method on the CelebA dataset with different mask rates. ↓ denotes that lower is better and ↑ indicates that higher is better. The best result is highlighted in **bold** and the second-best result is marked with underline.

Dataset		CelebA			
**Mask Ratio**		**(0.1, 0.2]**	**(0.2, 0.3]**	**(0.3, 0.4]**	**(0.4, 0.5]**
FID (↓)	EC	**2.0626**	**2.8117**	**4.0842**	**6.0656**
	RFR	3.2892	4.8099	6.9820	9.8065
	CTSDG	3.9021	6.7093	10.5437	15.1646
	SPL	2.2515	3.1305	4.5734	6.3852
	Ours	2.3552	3.1706	4.6410	7.1648
LPIPS (↓)	EC	0.026	0.0468	0.0721	0.1032
	RFR	0.0232	0.0416	0.0641	**0.0908**
	CTSDG	0.0293	0.0551	0.0858	0.1208
	SPL	0.0359	0.0578	0.0839	0.1142
	Ours	**0.0195**	**0.0385**	**0.0638**	0.0962
SSIM (↑)	EC	0.952	0.9148	0.8712	0.821
	RFR	0.9583	0.923	0.8813	0.834
	CTSDG	0.9533	0.9146	0.8697	0.8199
	SPL	**0.9632**	**0.9358**	**0.9022**	**0.8624**
	Ours	0.9613	0.9285	0.8892	0.8445
PSNR (↑)	EC	32.0645	28.6826	26.1033	24.0045
	RFR	32.8072	29.2923	26.6869	24.6201
	CTSDG	31.996	28.4897	25.9326	23.9307
	SPL	**33.7915**	**30.611**	**28.0283**	**25.8719**
	Ours	33.356	29.7005	26.9822	24.8199

**Table 4:** Quantitative comparison between EC, CTSDG, CR-Fill, SPL, and our method on the Places2 dataset with different mask rates. ↓ denotes that lower is better and ↑ indicates that higher is better. The best result is highlighted in **bold** and the second-best result is marked with underline.

Dataset		Places2			
**Mask Ratio**		**(0.1, 0.2]**	**(0.2, 0.3]**	**(0.3, 0.4]**	**(0.4, 0.5]**
FID (↓)	EC	1.4810	3.3814	6.2819	10.7867
	CTSDG	1.1672	3.3474	7.3858	14.0385
	CR-Fill	0.9558	2.2416	4.3691	**7.7783**
	SPL	**0.6680**	**1.8749**	**4.0821**	7.7864
	Ours	0.6796	1.9225	4.8417	10.5155
LPIPS (↓)	EC	0.0515	0.0897	0.1323	0.1804
	CTSDG	0.048	0.0925	0.1444	0.2021
	CR-Fill	0.0444	0.0808	0.1219	0.1689
	SPL	0.0373	0.0726	0.114	**0.1618**
	Ours	**0.0365**	**0.0709**	**0.1139**	0.1657
SSIM (↑)	EC	0.9225	0.8654	0.8039	0.737
	CTSDG	0.935	0.8795	0.8186	0.7522
	CR-Fill	0.9325	0.8784	0.8193	0.7542
	SPL	**0.9547**	**0.9128**	**0.8643**	**0.8089**
	Ours	0.9419	0.8929	0.8378	0.777
PSNR (↑)	EC	27.9966	24.9664	22.826	21.1286
	CTSDG	29.0271	25.6747	23.3848	21.6154
	CR-Fill	28.5685	25.1761	22.8066	20.95
	SPL	**31.2566**	**27.7344**	**25.2727**	**23.3253**
	Ours	29.8566	26.4925	24.1477	22.3093

**Table 5:** Quantitative comparison ablation experiment on the Paris StreetView dataset with different combinations of the proposed DDCM and ESA. **✓** and **✗** indicate the presence of the corresponding module. ↓ indicates that lower is better and ↑ indicates that higher is better. The best result is highlighted in **bold**.

Dataset			Paris Streetview			
**DDCM**		**ESA**	**Average**	**Average**	**Average**	**Average**
**Dilated**	**Dense**		**FID**	**LPIPS**	**SSIM**	**PSNR**
**Conv.**	**Block**		(↓)	(↓)	(↑)	(↑)
**✗**	**✗**	**✗**	42.6749	0.1005	0.8690	27.5524
**✗**	**✗**	**✓**	41.1195	0.0971	0.8713	27.6425
**✗**	**✓**	**✗**	40.8669	0.0971	0.8717	27.6685
**✗**	**✓**	**✓**	39.8682	0.0963	0.8740	27.7794
**✓**	**✗**	**✗**	39.4503	0.0920	0.8737	27.8604
**✓**	**✗**	**✓**	38.8718	0.0911	0.8745	27.9010
**✓**	**✓**	**✗**	38.0788	0.0903	0.8771	28.0407
**✓**	**✓**	**✓**	**35.7588**	**0.0869**	**0.8796**	**28.1083**

**Table 6:** Number of parameters and complexity of different combinations of the proposed DDCM and ESA. **✓** and **✗** indicate the presence of the corresponding module.

DDCM		ESA	Number of	Operational
Dilated Conv.	Dense Block		Model Parameters	Memory
**✗**	**✗**	**✗**	418,763	286.1
**✗**	**✗**	**✓**	695,243	300.9
**✗**	**✓**	**✗**	624,395	287.4
**✗**	**✓**	**✓**	900,875	301.2
**✓**	**✗**	**✗**	418,763	287
**✓**	**✗**	**✓**	695,243	300.8
**✓**	**✓**	**✗**	624,395	287.8
**✓**	**✓**	**✓**	900,875	301.4

## Data Availability

The data presented in this study are openly available.
